# Combined in-stent radiofrequency ablation and cholangioscopy-guided laser ablation for complex recurrent biliary obstruction

**DOI:** 10.1055/a-2749-3047

**Published:** 2025-12-08

**Authors:** Yan Liang, Yuan-Yuan Li, Xiao-Chun Yin, Qing-Hua Ji, Ya-Dong Feng

**Affiliations:** 1 162752 Department of Gastroenterology, Zhongda Hospital, Southeast University, Nanjing, China; 2 12582 School of Public Health, Soochow University, Suzhou, China

A 77-year-old woman with malignant hilar biliary obstruction (MHBO) secondary to unresectable gallbladder carcinoma presented with jaundice and fever. Coronal computed tomography reconstruction revealed a self-expandable metal stent (SEMS) in the right intrahepatic bile duct, with absent pneumobilia and dilated left intrahepatic bile ducts ( Fig. 1 ). Cholangioscopy identified tumor ingrowth as the causative stenosis, which was managed with repeated in-stent radiofrequency ablation (IS-RFA) in a stepwise manner ( Fig. 2 **a, b** ). A balloon catheter was then used to remove the resultant necrotic tissue. Although the left hepatic duct orifice was not directly visible on cholangioscopy, purulent bile was seen flowing out from the left side of the bile duct, serving as a critical clue to its location. Cholangioscopy-guided holmium laser dissection and ablation addressed the stricture and barrier of the SEMS at the left hepatic duct orifice, thereby alleviating the stricture, expanding the stent mesh, and allowing the cholangioscope to pass through the SEMS into the left hepatic duct ( Fig. 2 **c–e** ). After stone extraction from the left hepatic duct using a basket, bilateral plastic stents were placed to establish effective drainage ( Fig. 2 **f–i** , Fig. 3 ). The patient remained afebrile postoperatively, and the bilirubin level normalized within 1 month ( Video 1 ).

**Fig. 1:**
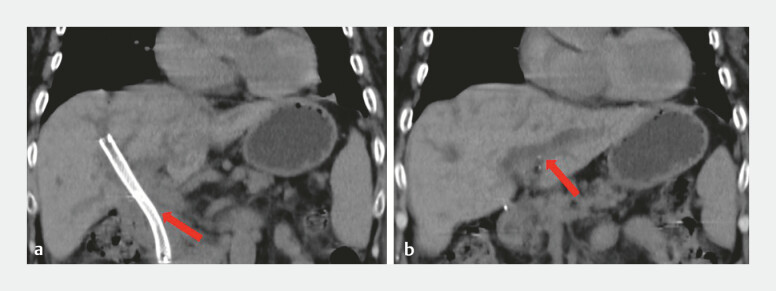
CT imaging prior to ERCP. **a** Coronal CT reconstruction revealing a SEMS in the right intrahepatic bile duct with absent pneumobilia. **b** Coronal CT reconstruction revealing dilated left hepatic ducts. CT, computed tomography; ERCP, endoscopic retrograde cholangiopancreatography; SEMS, self-expandable metal stent.

**Fig. 2:**
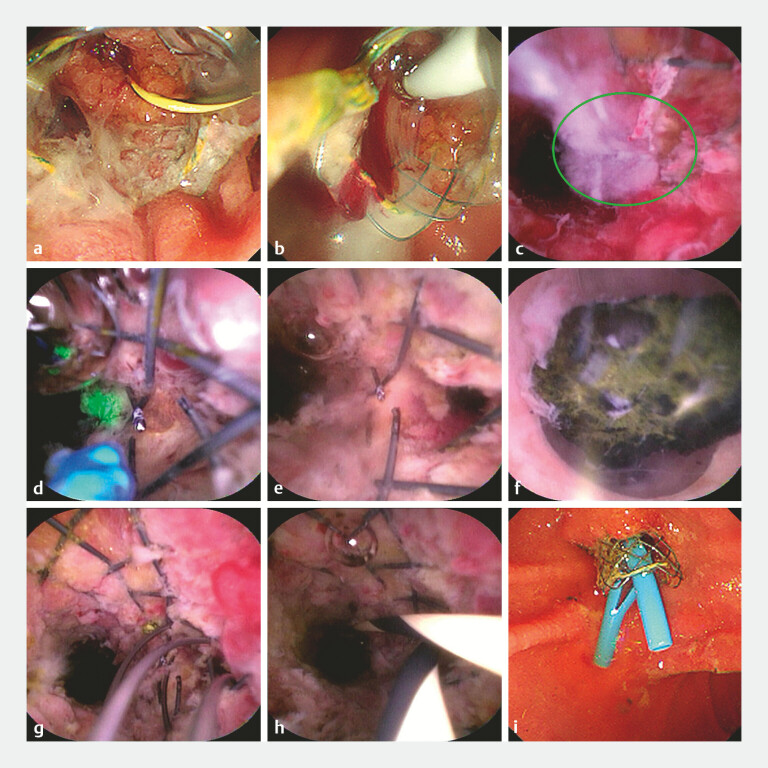
Management of a complex hilar obstruction with IS-RFA, holmium laser ablation, stone extraction, and bilateral stenting. **a** Cholangioscopy revealing purulent bile secondary to tumor ingrowth. **b** A stepwise IS-RFA procedure to alleviate stent re-occlusion. **c** The left hepatic orifice (within the green circle) was identified by the drainage of purulent bile. **d** Recanalization of the left hepatic orifice by cholangioscopy-guided holmium laser ablation of obstructing tissue and stent mesh. **e** Clear view of the left hepatic orifice following holmium laser ablation. **f** Calculus within the left hepatic duct. **g** Cholangioscopy-guided basket removal of left hepatic duct stones. **h** Guidewire positioned in the right and left hepatic ducts. **i** Bilateral plastic stents were deployed in the hepatic ducts. IS-RFA, in-stent radiofrequency ablation.

**Fig. 3:**
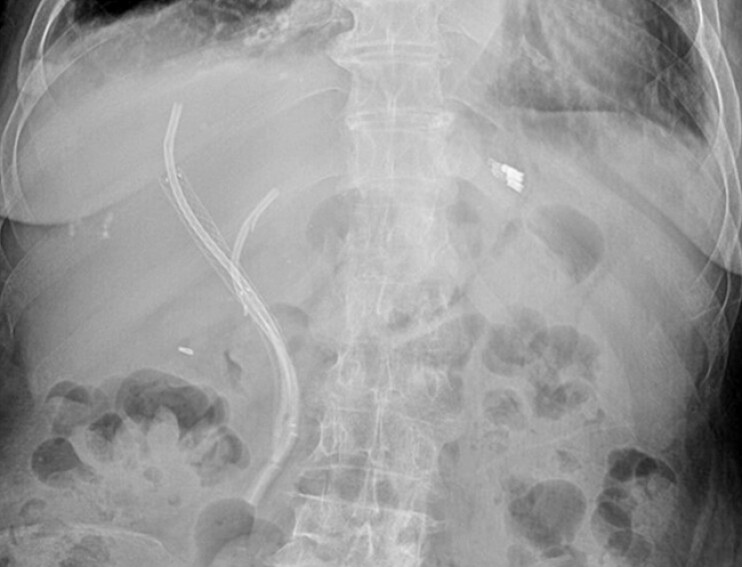
Abdominal plain film imaging after ERCP. Abdominal X-ray showing two 8.5-French plastic stents in situ within the left and right hepatic ducts. ERCP, ERCP, endoscopic retrograde cholangiopancreatography.

Cholangioscopy-guided holmium laser ablation at the left hepatic duct orifice.Video 1

IS-RFA 1 and laser ablation 2 3 are safe and feasible salvage therapies in pancreatobiliary strictures and neoplasia. This case demonstrates a novel application of cholangioscopy-guided holmium laser ablation for stent mesh widening – a technique that may significantly improve the feasibility of stent-in-stent procedures in MHBO. Our work has pioneered a combined IS-RFA and holmium laser ablation approach for complex recurrent biliary obstruction, yielding satisfactory clinical outcomes.

Endoscopy_UCTN_Code_TTT_1AR_2AF

## Notes

Endoscopy E-Videos https://eref.thieme.de/e-videos
*E-Videos* is an open access online section of the journal *Endoscopy* , reporting on interesting cases and new techniques in gastroenterological endoscopy. All papers include a high-quality video and are published with a Creative Commons CC-BY license. Endoscopy E-Videos qualify for HINARI discounts and waivers and eligibility is automatically checked during the submission process. We grant 100% waivers to articles whose corresponding authors are based in Group A countries and 50% waivers to those who are based in Group B countries as classified by Research4Life (see: https://www.research4life.org/access/eligibility/ ). This section has its own submission website at https://mc.manuscriptcentral.com/e-videos .
